# Supplementary material for: Detection of a historic reservoir of bedaquiline/clofazimine resistance-associated variants in Mycobacterium tuberculosis
Source: Genome Med. 2024 Feb 19;16:34. doi: 10.1186/s13073-024-01289-5 (PMC10877763; doi:10.1186/s13073-024-01289-5)
Supplement: Supplementary file 2 — Additional file 2: Fig S1. Distribution of difference (in decimal years) of collection date to release date for sequences in the lineage 2 (L2) and lineage 4 (L4) dataset. For lineage 4, having excluded samples from three eighteenth century mummies, an average of 6.9 years (0.6–19.1 years 95% CI) was obtained. For lineage 2, an average lag-time of 4.7 years (0.5–12.6 years 95% CI) was obtained. Fig S2. Maximum likelihood phylogenetic tree of the lineage 2 (a) and lineage 4 (b) datasets. Tip colours provide the country of sample collection and outer bars give the resistance to four antimycobacterial drugs: fluroquinolones (FLQ), isoniazid (INH), kanamycin (KAN) and rifampicin (RMP). Fig S3. Number (count) of mmpR5 variants split by predicted phenotype (x-axis) for each geographic region included in each of the lineage 2 (a) and lineage 4 b) datasets. Fig S4. Co-occurrence count of mmpR5 variant phenotype predictions (x-axis) and resistance status a) and genotypic resistances b) for the lineage 2 dataset. Fig S5. Co-occurrence count of mmpR5 variant phenotype predictions (x-axis) and resistance status a) and genotypic resistances b) for the lineage 4 dataset. Fig S6. Contingency tables and χ2 results for the distribution of susceptible, MDR and XDR Mtb amongst strains carrying variants in mmpR5 in the lineage 2 dataset. Plots provide the squared standardised residuals contributing to the rejection of the null hypothesis. The colour intensity and size of the circle is proportion to the contribution with positive displayed in blue and negative in red. Fig S7. Contingency tables and χ2 results for the distribution of susceptible, MDR and XDR Mtb amongst strains carrying variants in mmpR5 in the lineage 4 dataset. Plots provide the squared standardised residuals contributing to the rejection of the null hypothesis. The colour intensity and size of the circle is proportion to the contribution with positive displayed in blue and negative in red. Fig S8. Phylogenetic distrib [file 13073_2024_1289_MOESM2_ESM.pdf]

Supplementary Material

Detection of a historic reservoir of bedaquiline / clofazimine resistance associated variants in *Mycobacterium tuberculosis*

Camus Nimmo[1,2,3] camus.nimmo@crick.ac.uk

Arturo Torres Ortiz[1,4] a.ortiz@ucl.ac.uk

Cedric C.S. Tan[1] cedric.tan.18@ucl.ac.uk

Juanita Pang[1,2] juanita.pang.16@ucl.ac.uk

Mislav Acman[1] mislav.acman.17@alumni.ucl.ac.uk

James Millard[3,5,6] james.millard@ahri.org

Nesri Padayatchi[7] Nesri.Padayatchi@caprisa.org

Alison Grant[3,8] alison.grant@lshtm.ac.uk

Max O'Donnell[7,9] mo2130@cumc.columbia.edu

Alex Pym[3] APym@its.jnj.com

Ola B Brynildsrud[10] ola.brynildsrud@fhi.no

Vegard Eldholm[10] elve@fhi.no

Louis Grandjean[2,11,12] l.grandjean@ucl.ac.uk

Xavier Didelot[13] xavier.didelot@gmail.com

François Balloux[1*] f.balloux@ucl.ac.uk

Lucy van Dorp[1*] lucy.dorp.12@ucl.ac.uk

*These authors contributed equally.

Correspondence: Camus Nimmo (camus.nimmo@crick.ac.uk), François Balloux (f.balloux@ucl.ac.uk) and Lucy van Dorp (lucy.dorp.12@ucl.ac.uk)

1. UCL Genetics Institute, University College London, Darwin Building, Gower Street, London, WC1E 6BT, UK (Correspondence)
2. Division of Infection and Immunity, University College London, London, UK
3. Africa Health Research Institute, Durban, South Africa
4. Department of Medicine, Imperial College, London, UK
5. Wellcome Trust Liverpool Glasgow Centre for Global Health Research, Liverpool, UK
6. Institute of Infection and Global Health, University of Liverpool, Liverpool, UK

7. CAPRISA MRC-HIV-TB Pathogenesis and Treatment Research Unit, Durban, South Africa
8. TB Centre, London School of Hygiene & Tropical Medicine, London, UK
9. Department of Medicine & Epidemiology, Columbia University Irving Medical Center, New York, NY, USA
10. Division of Infectious Diseases and Environmental Health, Norwegian Institute of Public Health, Oslo, Norway
11. Laboratorio de Investigacion y Enfermedades Infecciosas/Universidad Peruana Cayetano Heredia, Lima, Peru
12. Department of Infection, Immunity and Inflammation, Institute of Child Health, University College London, London, UK
13. School of Life Sciences and Department of Statistics, University of Warwick, Coventry, UK

Supplementary Figures
Pages 3-15

Supplementary Tables
Pages 16-23
Table S1, S7 and S8 are available as external excel spreadsheets.

Supplementary Note
Pages 24-28

References
Pages 29-30

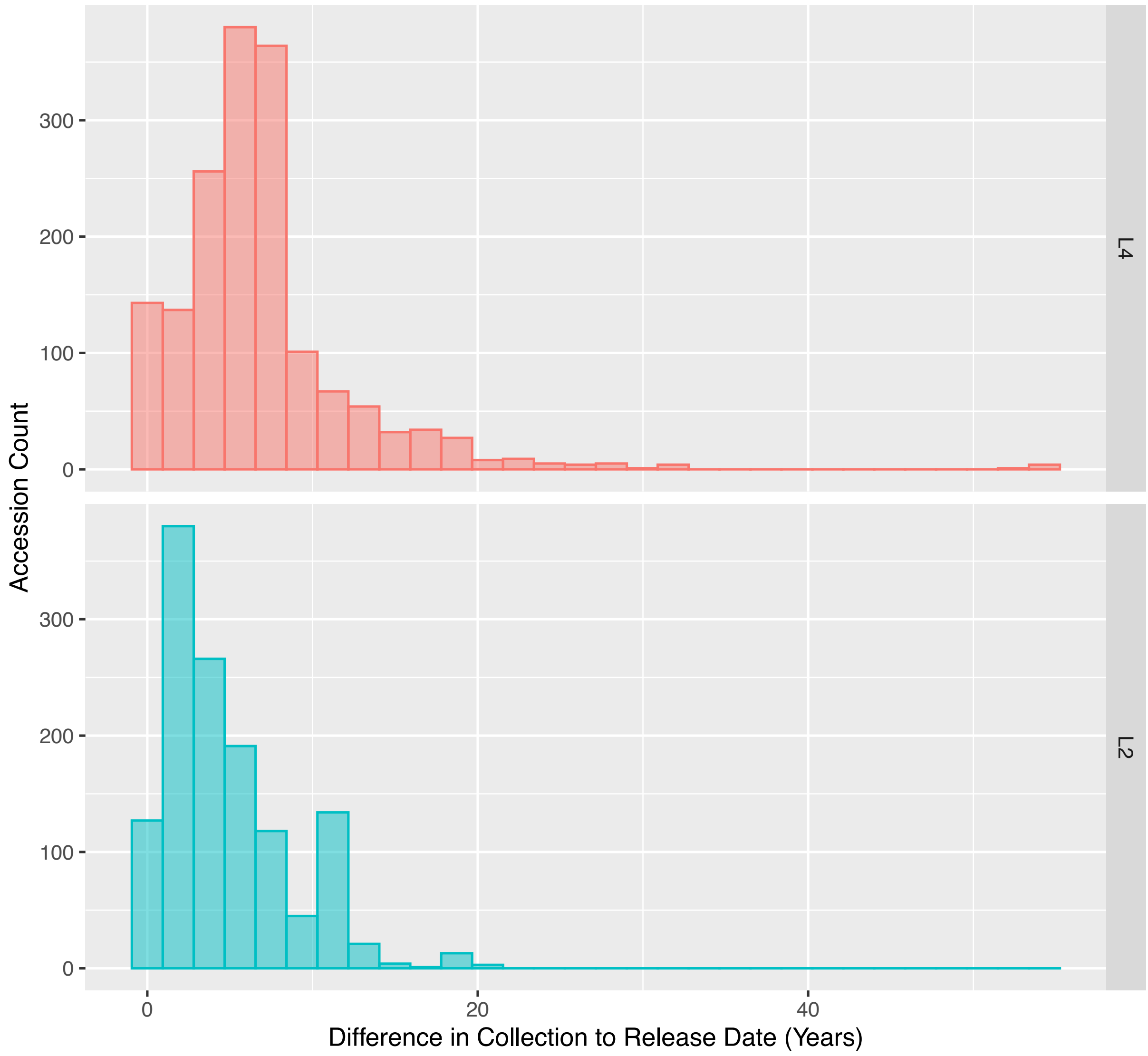

Fig S1: Distribution of difference (in decimal years) of collection date to release date for sequences in the lineage 2 (L2) and lineage 4 (L4) dataset. For lineage 4, having excluded samples from three 18th century mummies, an average of 6.9 years (0.6-19.1 years 95% CI) was obtained. For lineage 2, an average lag-time of 4.7 years (0.5–12.6 years 95% CI) was obtained.

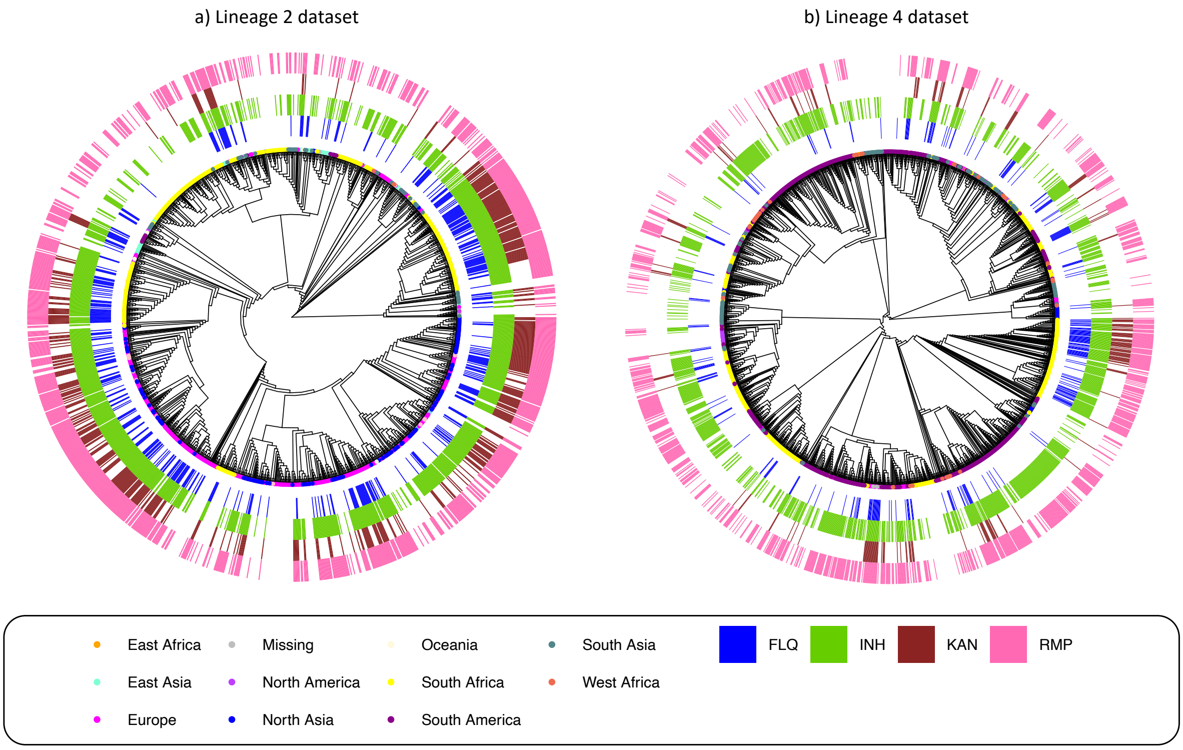

Fig S2: Maximum likelihood phylogenetic tree of the lineage 2 (a) and lineage 4 (b) datasets. Tip colours provide the country of sample collection and outer bars give the resistance to four antimycobacterial drugs: fluroquinolones (FLQ), isoniazid (INH), kanamycin (KAN) and rifampicin (RMP).

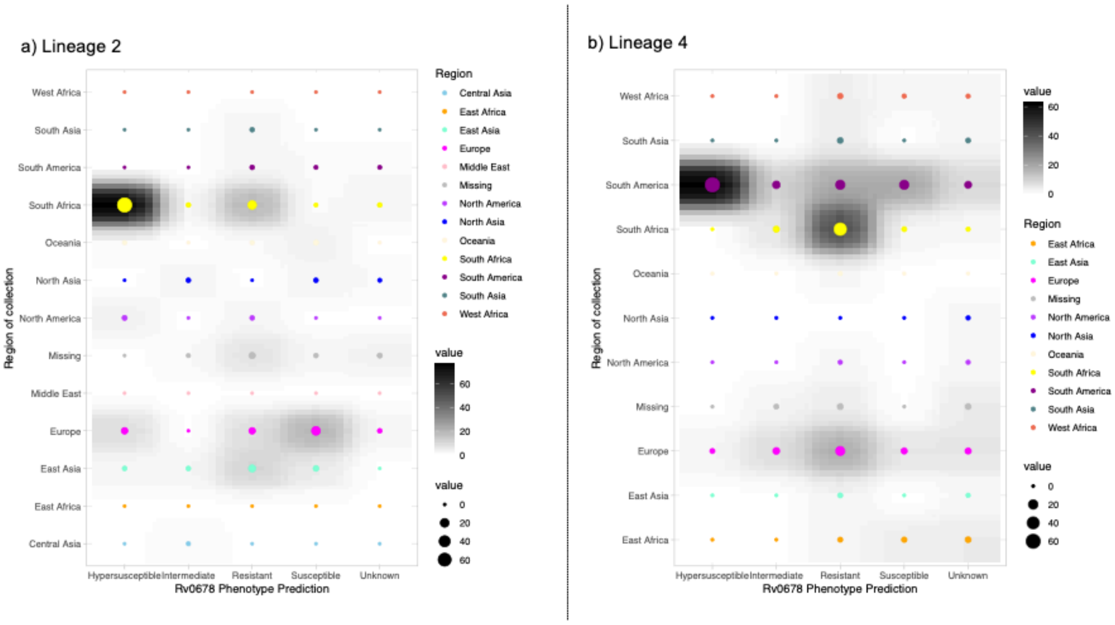

Fig S3: Number (count) of *mmpR5* variants split by predicted phenotype (x-axis) for each geographic region included in each of the lineage 2 (a) and lineage 4 b) datasets.

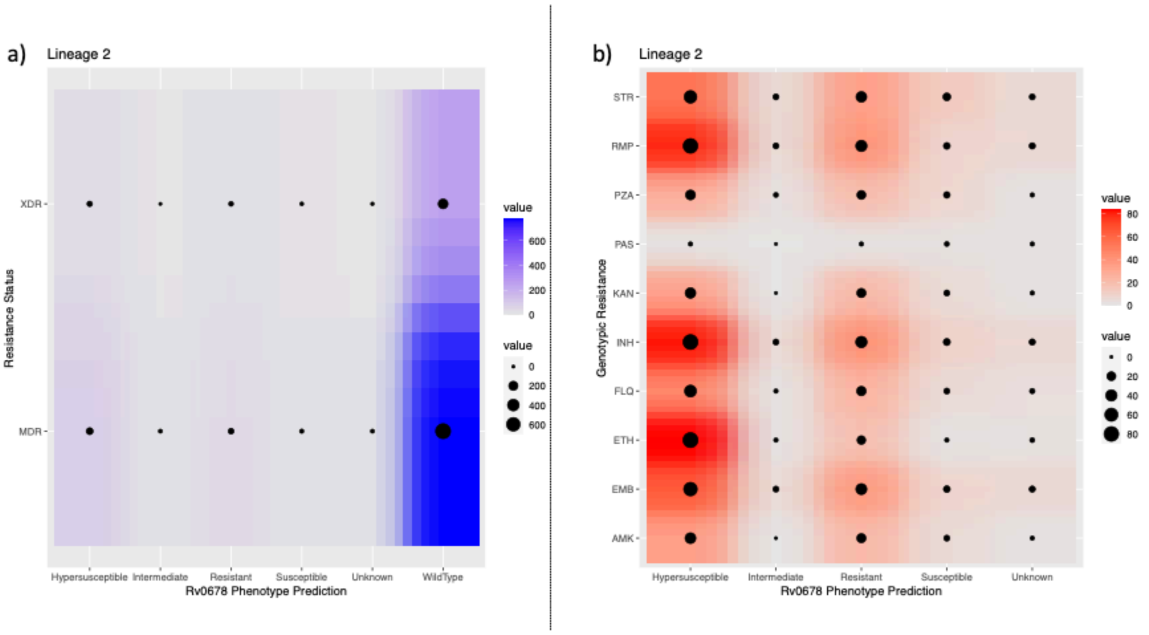

Fig S4: Co-occurrence count of *mmpR5* variant phenotype predictions (x-axis) and resistance status a) and genotypic resistances b) for the lineage 2 dataset.

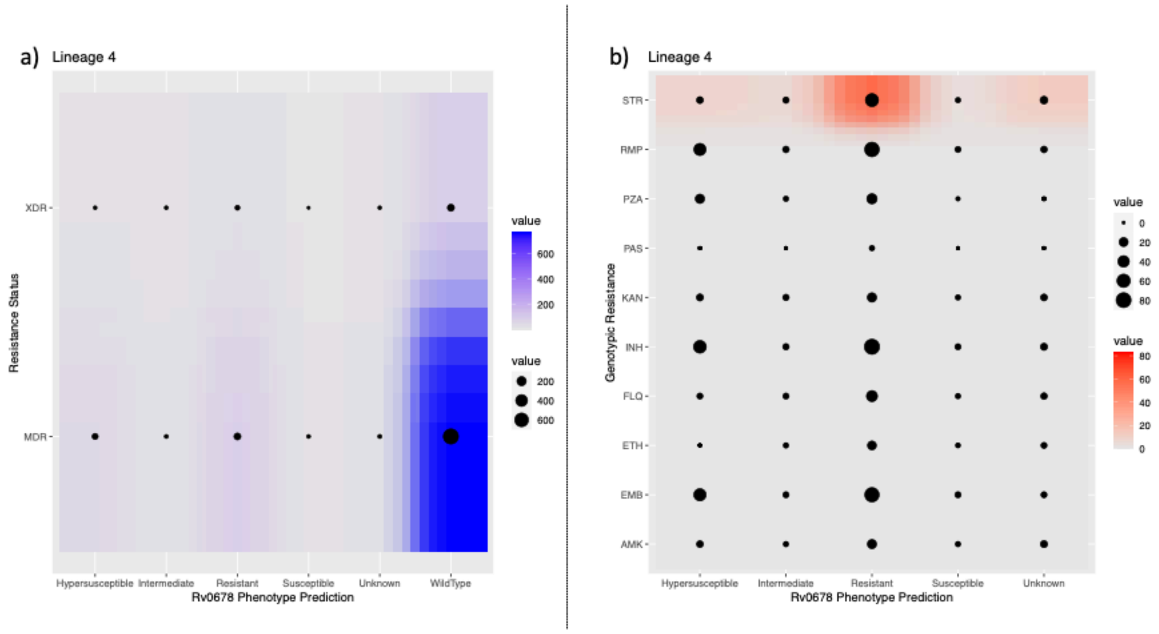

Fig S5: Co-occurrence count of *mmpR5* variant phenotype predictions (x-axis) and resistance status a) and genotypic resistances b) for the lineage 4 dataset.

Classification	MDR	S	XDR
Hypersusceptible	48	7	30
Intermediate	5	3	0
Resistant	24	11	16
Susceptible	5	21	3
Unknown	4	2	1
Wildtype	530	330	245

Chi=51.875, df=10, p=1.2e-07

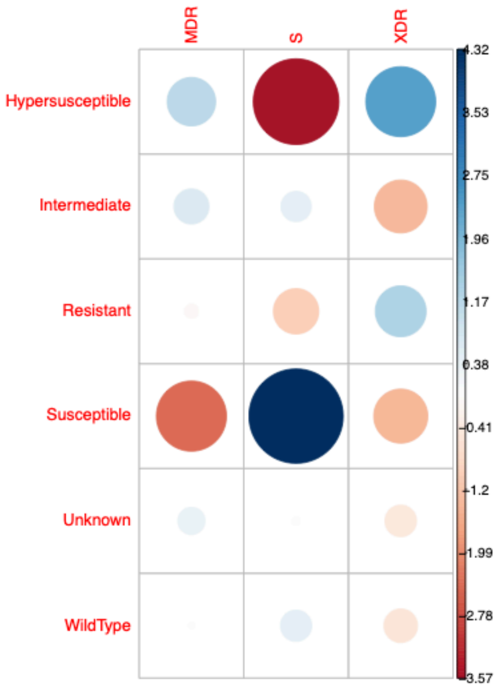

Fig S6: Contingency tables and χ^2 results for the distribution of susceptible, MDR and XDR *Mtb* amongst strains carrying variants in *mmpR5* in the lineage 2 dataset. Plots provide the squared standardised residuals contributing to the rejection of the null hypothesis. The colour intensity and size of the circle is proportion to the contribution with positive displayed in blue and negative in red.

Classification	MDR	S	XDR
Hypersusceptible	43	11	4
Intermediate	1	17	5
Resistant	57	11	19
Susceptible	3	25	2
Unknown	3	10	4
Wildtype	689	702	81

Chi=127.83, df=10, p<2.2e-16

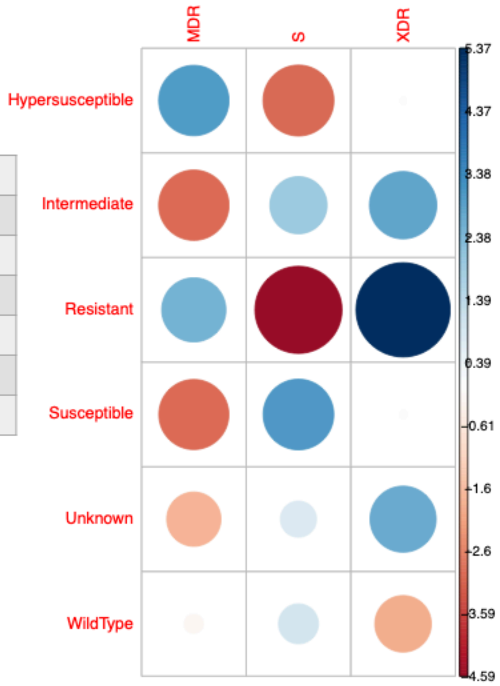

Fig S7: Contingency tables and χ^2 results for the distribution of susceptible, MDR and XDR *Mtb* amongst strains carrying variants in *mmpR5* in the lineage 4 dataset. Plots provide the squared standardised residuals contributing to the rejection of the null hypothesis. The colour intensity and size of the circle is proportion to the contribution with positive displayed in blue and negative in red.

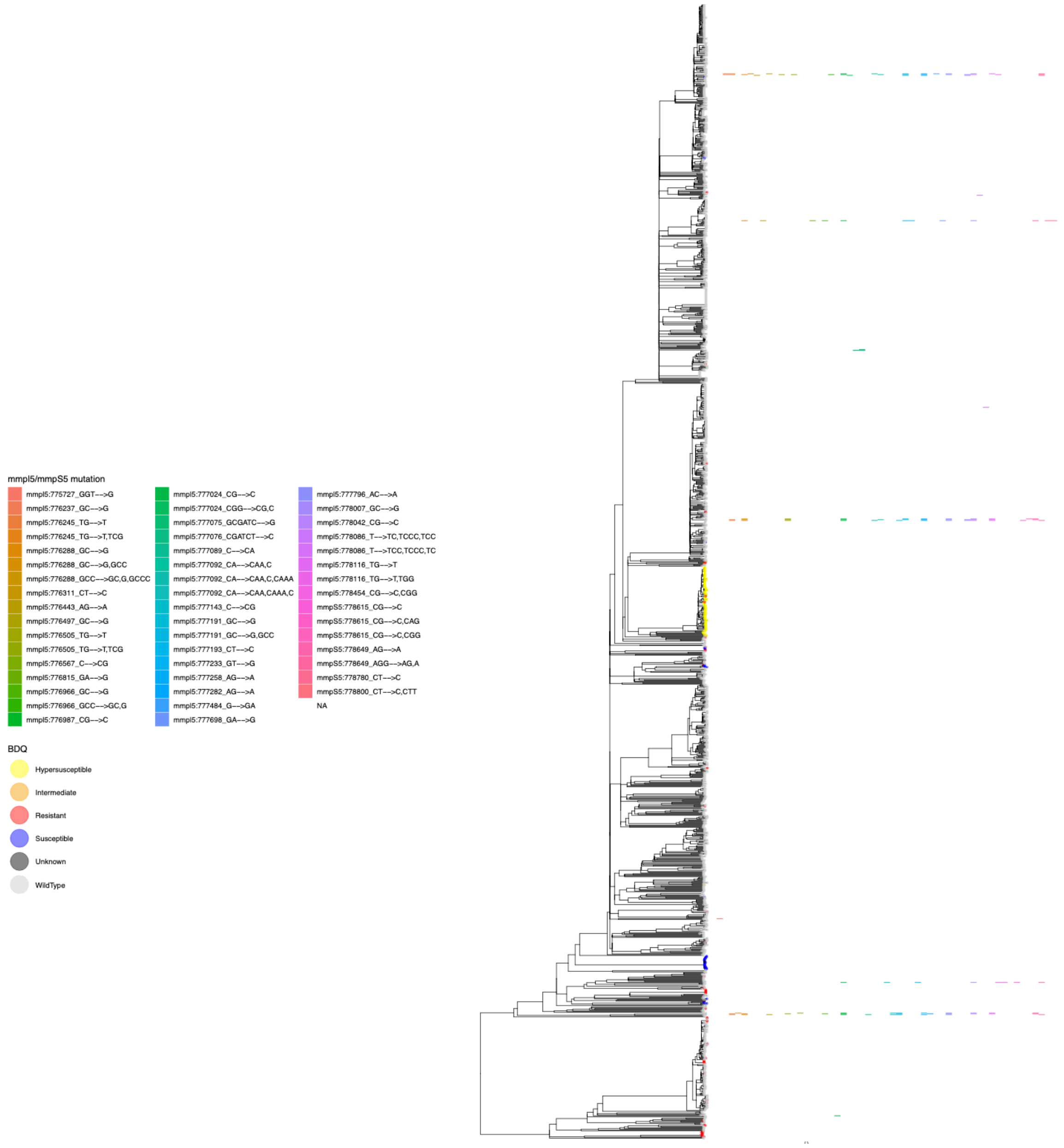

Fig S8: Phylogenetic distribution of LOF mutations identified in *mmpL5* and *mmpS5* identified in L2 isolates. Phylogeny is provided with tip colours according to inferred bedaquiline resistance status. Heatmap provides colour for presence of a mutation as ordered by the vertical columns.

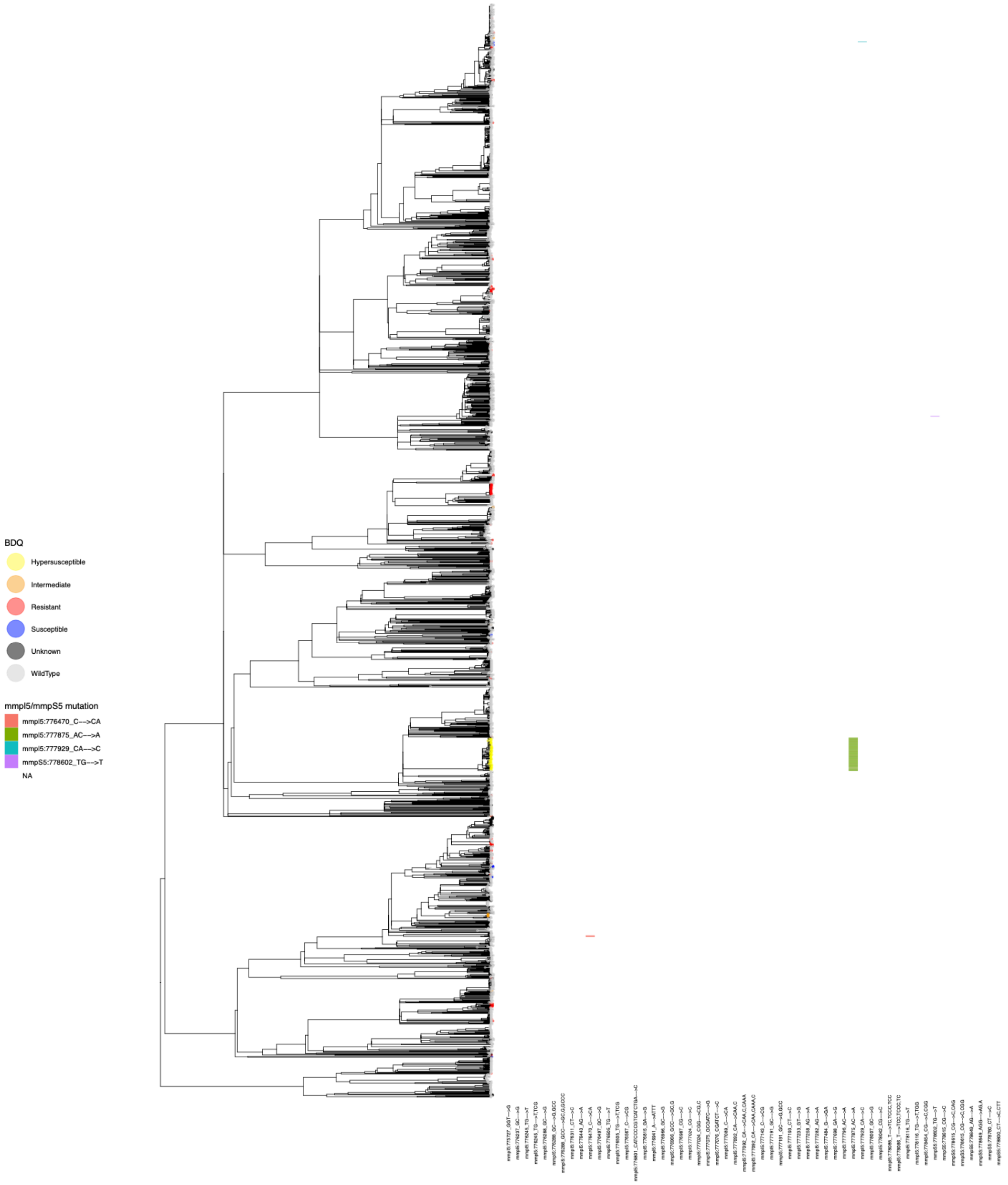

Fig S9: Phylogenetic distribution of LOF mutations identified in *mmpL5* and *mmpS5* identified in L4 isolates. Phylogeny is provided with tip colours according to inferred bedaquiline resistance status. Heatmap provides colour for presence of a mutation as ordered by the vertical columns.

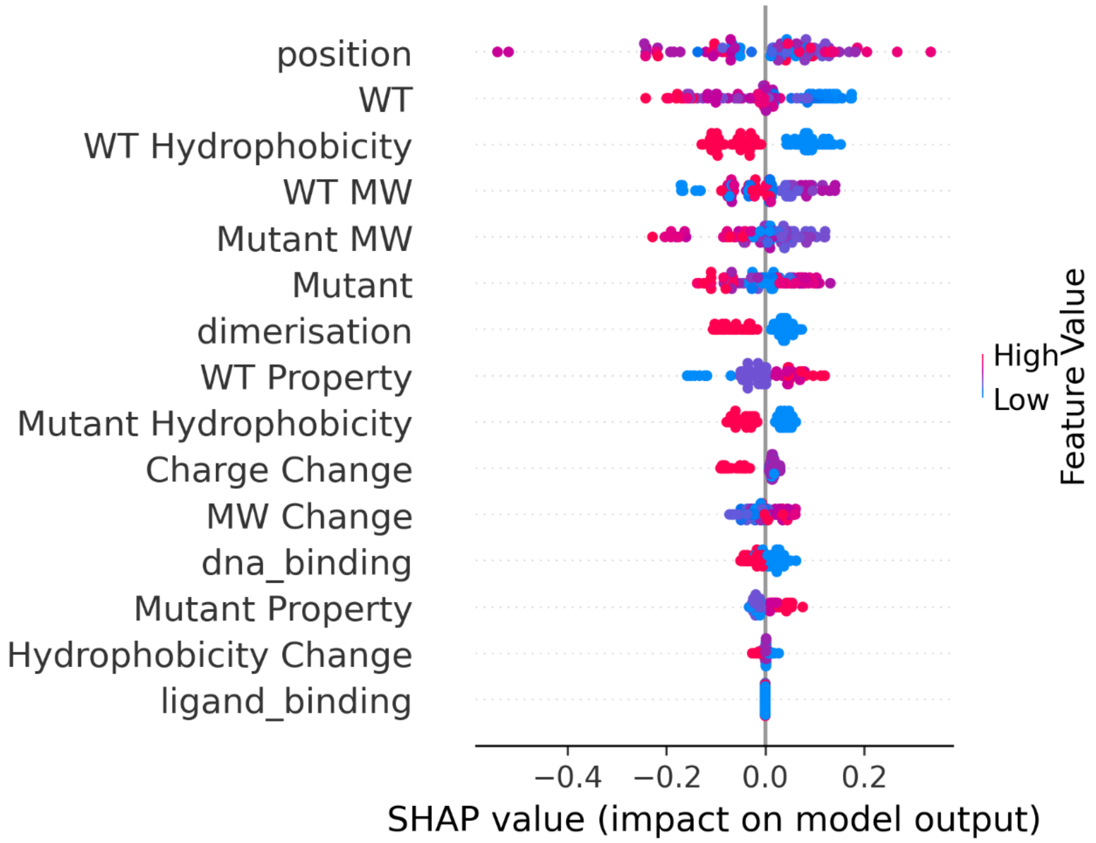

Fig S10: Summary plot of SHAP values. Each point represents the SHAP value of a single prediction for a particular feature. Points are stacked vertically using density estimation. 'WT', 'mutant' and 'MW' denotes the wild type amino acid, amino acid variant and molecular weight (Da) respectively. 'Property' refers to whether an amino acid was non-polar, polar, positively charged or negatively charged. 'Ligand_binding', 'dna_binding' and 'dimerisation' refer to whether the amino acid residue is involved in ligand binding, DNA binding or dimerisation. 'Position' refers to the integer 5'-3' position of the variant. Positive SHAP values imply an increase in the predicted probability of resistance due to the presence of the feature.

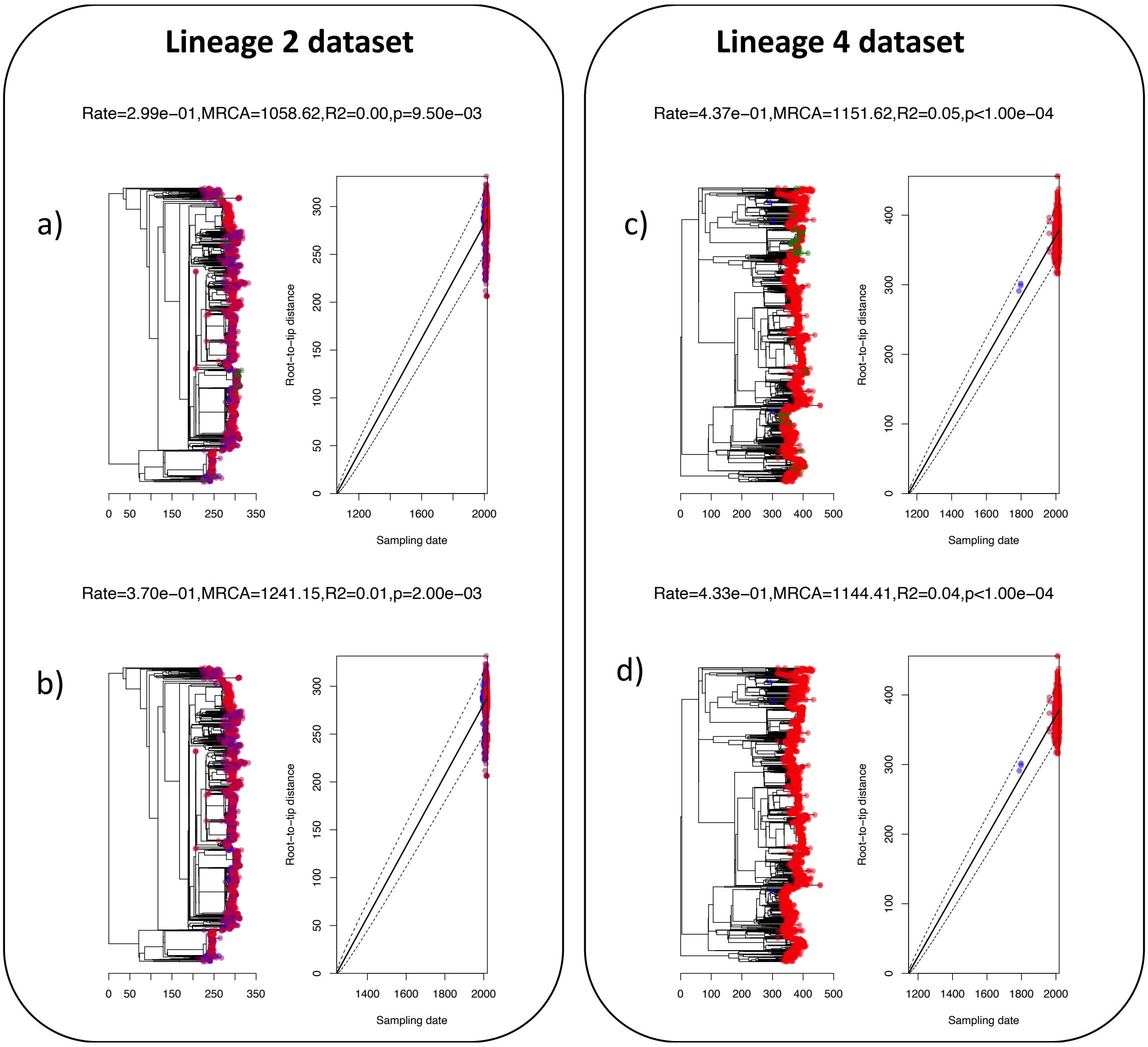

Fig S11: Linear regressions of root-to-tip distance (y-axis) versus sampling dates (x-axis) for global *Mtb* datasets; lineage 2 (a-b) and lineage 4 (c-d). Regressions are performed both without (a-c) and with (b-d) imputation of missing dates (see Methods). Here, the *p*-value (tip-randomisation test) is calculated by fitting a linear regression to the root to tip distance vs sampling date for 10,000 randomisations and adding the number of randomised fits that present a better regression coefficient than the real data (divided by 10,000).

a) Lineage 2

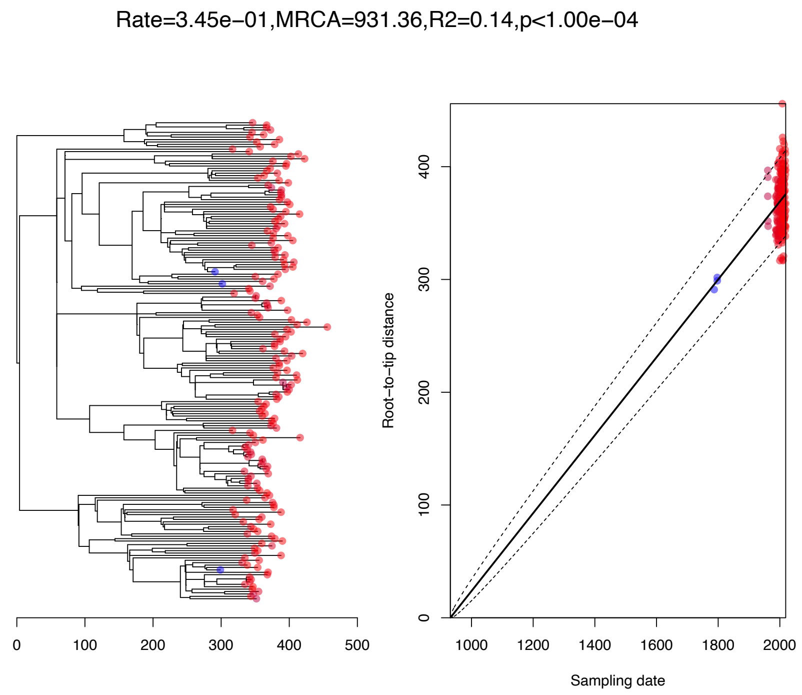

b) Lineage 4

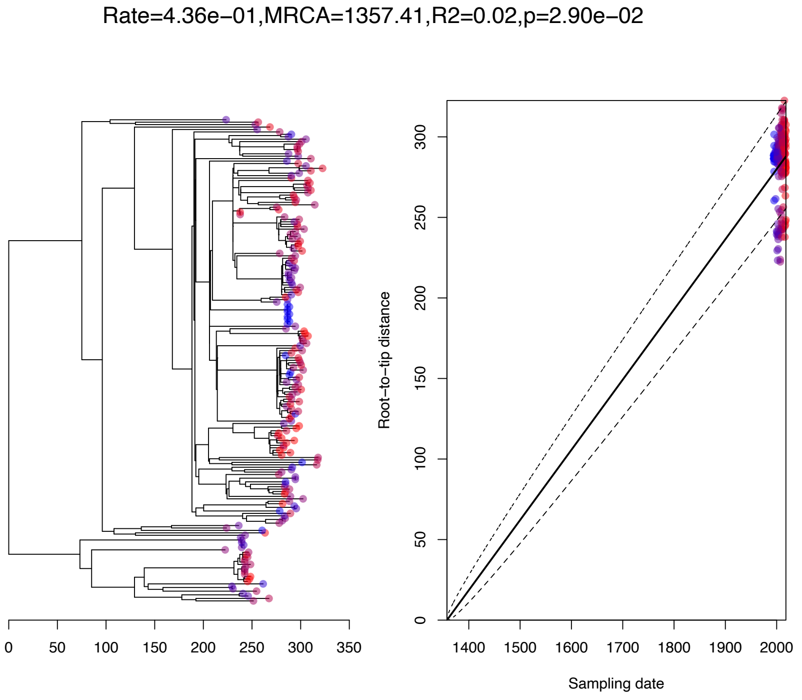

Fig S12: Sub-sampled datasets temporal regression. The lineage 2 data covered 24 years of molecular evolution. The lineage 4 dataset comprised 232 years of evolution.

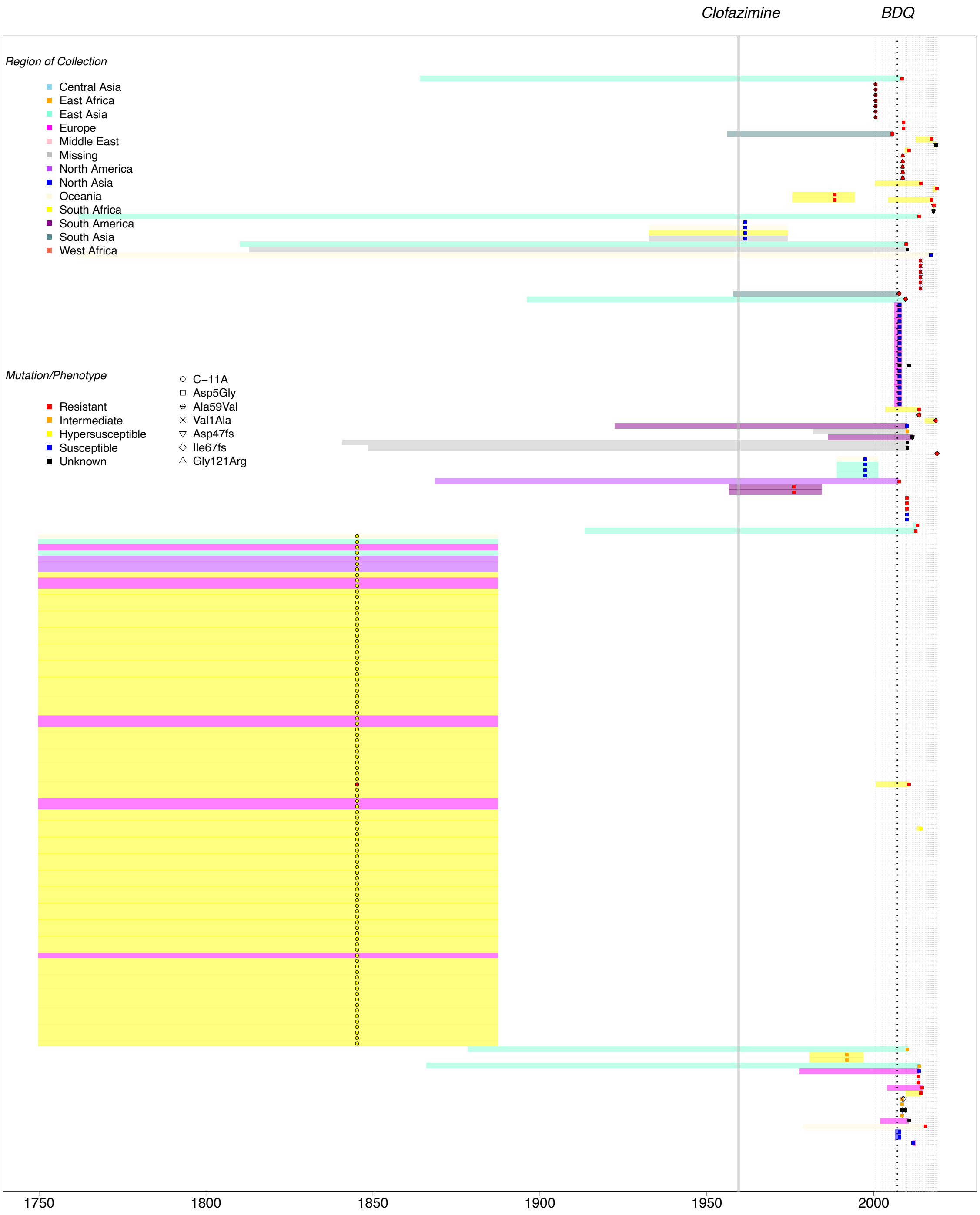

Fig S13: Full mutational timeline for the estimated date of emergence (x-axis) and confidence intervals of nodes with descendent tips carrying nonsynonymous variants in *mmpR5* in the lineage 2 dataset. All nonsynonymous variants are depicted. Confidence bars are coloured according to the region where the isolate was collected. Symbols provide the point estimates of the age of the node coloured by *mmpR5* predicted phenotype. Symbols are used for all mutations occurring in ≥5 isolates, in this case. Grey dashed lines provide the collection date of all sequenced isolates included in the analysis with *mmpR5* variants. Data available in Supplementary Table S7.

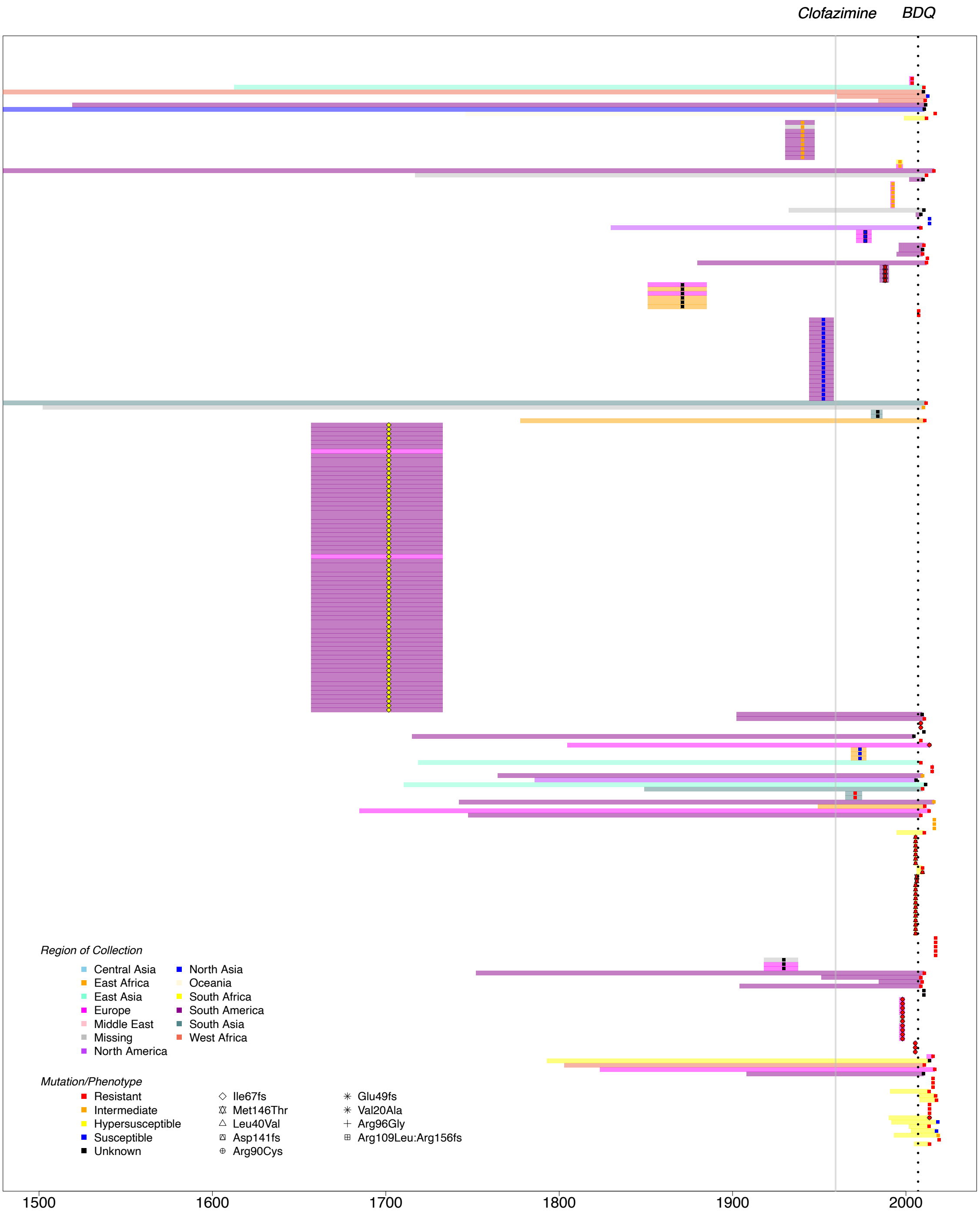

Fig S14: Full mutational timeline for the estimated date of emergence (x-axis) and confidence intervals of nodes with descendent tips carrying nonsynonymous variants in *mmpR5* in the lineage 4 dataset. All nonsynonymous variants are depicted. Confidence bars are coloured according to the region where the isolate was collected. Symbols provide the point estimates of the age of the node coloured by *mmpR5* predicted phenotype. Symbols are used for all mutations occurring in ≥5 isolates. Grey dashed lines provide the collection date of all sequenced isolates included in the analysis with *mmpR5* variants. Data available in Supplementary Table S7.

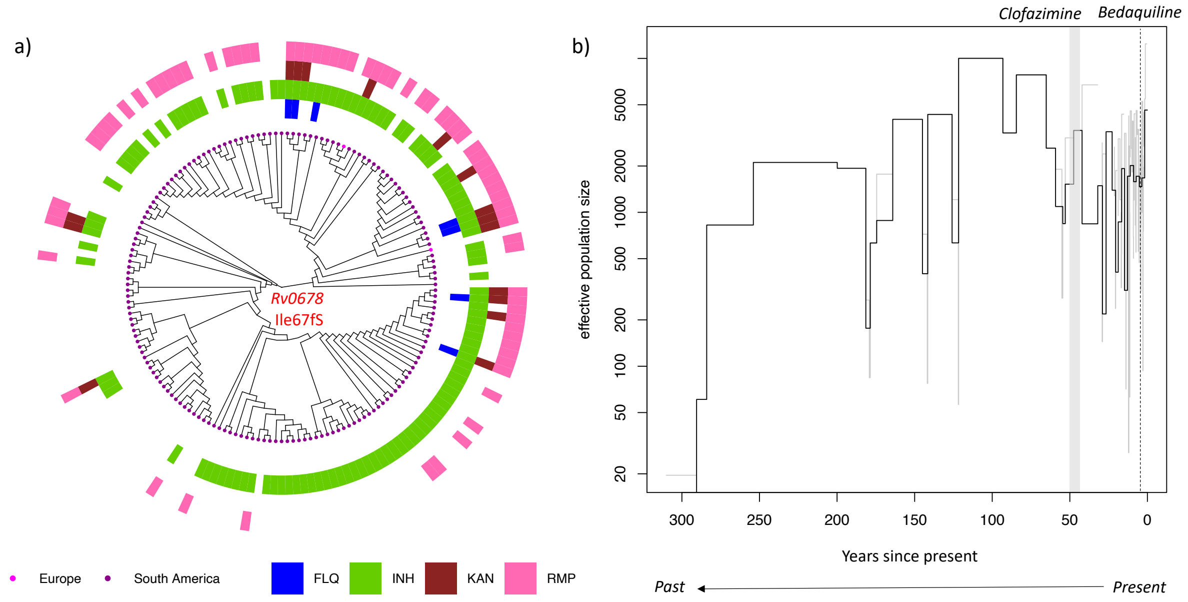

Fig S15: a) Lineage 4 Peruvian *mmpR5* Ile67fs + *mmpL5* Arg202fs carrying clade phylogeny, which has a tMRCA dating to 1702 (1657-1732). Phenotypic resistances for fluoroquinolones (FLQ), isoniazid (INH), kanamycin (KAN) and rifampicin (RMP) are provided as outer coloured rings. Most samples are from Peru (purple), though two samples are from Europe (Sweden and the Netherlands). b) Provides the generalized skyline plot estimate of effective population size through time based on the timed phylogeny of this clade. Grey lines provide the full skyline plot, black lines provide the coalescent intervals. The first clinical use of clofazimine and bedaquiline are provided by the axis at top.

Table S1 [external excel file]: Spreadsheet providing the whole genomes sequences included in both alignments with accession numbers and *mmpR5* variants annotated.

Table S2: Source of whole genome sequences included in the global lineage 2 and lineage 4 alignments following quality checks and as given in Table S1. The number in brackets designate those with *mmpR5* differing from wild type.

**Data source**	**Sequence count (with *mmpR5* variants)**	
	**Lineage 2**	**Lineage 4**
Base dataset	1016 (12)	1166 (20)
South Africa – our centre	155 (11)	243 (17)
Southern Africa – other studies (marked 'Additional' in Table S1)	238 (77)	426 (73)
Peru	11 (3)	266 (70)
BIGSI	73 (173)	40 (40)
PYSGI	18 (18)	24 (24)
Strain Bank sequences [1]	3 (0)	3 (0)
Total	1514 (294)	2168 (244)

Table S3: Classification of previously observed resistance associated with *mmpR5* variants identified in this study.

Observed resistance	Mutation	Reference
Resistant	Met1Ala	Bloemberg [1]
	Ser2Ile	Xu 2017 [2]
	Glu21Asp	Yang 2018 [3]
	Gln22Pro	Nimmo 2020 [4]
	Ser31Arg	Yang 2018 [3]
	Leu32Ser	CRyPTIC, Sonnenkalb 2023 [5]
	Thr33Ala	Ismail 2019 [6]
	Thr33Ser	Yang 2018 [4]
	Ala36Thr	Ismail 2019 [6]
	Cys46Arg	Ismail 2019 [6]
	Cys46fs	Nimmo 2020 [4]
	Pro48Leu	CRyPTIC, Sonnenkalb 2023 [5]
	Arg50Trp	Villellas 2017 [7], Sonnenkalb 2023 [5]
	Ser52Phe	Villellas 2017 [7]
	Ser53Pro	Ismail 2019 [6]
	Ala57Glu	Nimmo 2020 [8]
	Ala59Val	Villellas 2017 [7]
	Ala62Val	Villellas 2017 [7]
	Ile67Ser	Ismail 2018 [9]
	Ser68Gly	Andries 2014 [10]
	Arg72Trp	Ismail 2019 [6], Sonnenkalb 2023 [5]
	Arg72Thr	Nimmo 2020 [8]
	Leu74Val	CRyPTIC, Sonnenkalb 2023 [5]
	Leu74Pro	Ismail 2019 [6]
	Phe79Ser	Yang 2018 [3]
	Leu83Pro	Ismail 2019 [6]
	Ala84Val	Sonnenkalb 2023 [5]
	Asp88Arg	Nimmo 2020 [8]
	Tyr92Cys	Karmakar 2020 [11]
	Arg94Gln	Andries 2014 [10]
	Arg96Trp	CRyPTIC, Sonnenkalb 2023 [5]
	Arg96Gly	CRyPTIC, Sonnenkalb 2023 [5]
	Asn98Asp	Yang 2018 [3]
	Asn98fs	Ismail 2019 [6]
	Ala102Pro	Ismail 2019 [6]
	Ile108Val	Andres 2020 [12]
	Ala112Ser	Villellas 2017 [7]

	Leu117Arg	Xu 2017 [2]
	Ala118Thr	Nimmo 2020 [8]
	Gly121Arg	Nimmo 2020 [8]
	Leu122Pro	Nimmo 2020 [8]
	Arg123Lys	Yang 2018 [3]
	Arg135Gly	Ismail 2019 [6]
	Leu136Arg	Nimmo 2020 [8]
	Met139Ile	CRyPTIC, Sonnenkalb 2023 [5]
	Leu154Pro	Ismail 2018 [9]
	Met146Thr	Xu 2017[3], reported susceptible in Yang 2018
Intermediate	Pro14Leu	CRyPTIC, Sonnenkalb 2023 [5]
	Val20Gly	Nimmo 2020 [8]
	Val20Ala	Nimmo 2020 [8]
	Leu40Ser	Zimenkov 2017 [13]
	Ser53Leu	Pang 2017 [14]
	Ile67Val	Yang 2018 [3]
	Arg89Trp	Nimmo 2020 [8]
	Arg90Cys	Yang 2018 [3]
	Phe93Ser	Nimmo 2020 [8]
	Asn98Val	Zimenkov 2017 [13]
	Arg109Leu	Nimmo 2020 [4]
	Glu113Lys	Zimenkov 2017 [13]
	Gly121Glu	Zimenkov 2017 [13]
	Glu138Gly	Andries 2014 [10]
	Met139Thr	Veziris 2017 [15]
	Leu142Arg	Zimenkov 2017 [13]
Susceptible	Asp5Gly	Martinez 2018 [16]
	Met17Leu	Villellas 2017 [7]
	Met23Leu	Zimenkov 2017 [13]
	Met23Val	Martinez 2018 [16]
	Leu40Val	CRyPTIC, Sonnenkalb 2023 [5]
	Trp42Arg	Villellas 2017 [7]
	Glu55Asp	Martinez 2018 [16]
	Gly66Trp	Zimenkov 2017 [13]
	Gly78Arg	Zimenkov 2017 [13]
	Val85Ala	Zimenkov 2017 [13]
	Gly87Arg	Martinez 2018 [16]
	Asp88Gly	Yang 2018 [3]
	Phe93Leu	Nimmo 2020 [4]
	Ala101Thr	Villellas 2017 [7]
	Asp116His	Villellas 2017 [7]
	Arg135Trp	Zimenkov 2017 [13]

	Tyr145Phe	Villellas 2017 [7]
	Tyr157Asp	Pang 2017 [14]
	Ser158Arg	Villellas 2017 [7]
Hypersusceptible	C-11A	Villellas 2017 [7]
Undetermined frameshifts	Asp47fs	Sonnenkalb 2023 [5]
	Asp141fs	Rancoita 2018 [17] , reported susceptible (+ *mmpL5* I948V)

Table S4: Sequence data identified with *mmpR5* variants predating 2007. Dates flagged with an asterisk (*) indicate those dates which have been permuted using the metadata of all samples (see Est. column of Supplementary Table S1).

**Lineage 2**				
**Accession**	**Collection Year**	***mmpR5* variant**	**Predicted Phenotype**	**Location**
**SRR067502**	2000	Ala59Val	Resistant	USA
**SRR067488**	2000	Ala59Val	Resistant	Missing
**SRR067486**	2002	Ala59Val	Resistant	Missing
**SRR067484**	2003	Ala59Val	Resistant	Missing
**SRR070032**	2003	Ala59Val	Resistant	Missing
**SRR067534**	2004	Ala59Val	Resistant	Missing
**SRR6397632**	2005.5*	C-11A	Hypersusceptible	Canada
**SRR1013665**	2005	Asn98fs	Resistant	South Korea
**SRR6153262**	2006.5*	C-11A	Hypersusceptible	Canada
**SRR067468**	2006	Ala59Val	Resistant	Missing
**Lineage 4**				
**MDRDM260**	1999	Ile67fs + *mmpL5* R202fs	Hypersusceptible	Missing
**ERR028625**	1999	Val20Ala	Intermediate	Netherlands
**ERR025419**	1999	Val20Ala	Intermediate	Netherlands
**ERR025417**	1999	Val20Ala	Intermediate	Netherlands
**ERR025414**	1999	Val20Ala	Intermediate	Netherlands
**ERR025418**	1999	Val20Ala	Intermediate	Netherlands
**ERR028626**	1999	Val20Ala	Intermediate	Netherlands
**LE-006**	2003	Ile67fs + *mmpL5* R202fs	Hypersusceptible	Peru
**HO-109**	2003	Ile67fs + *mmpL5* R202fs	Hypersusceptible	Peru
**LN3756**	2004	Ile67fs + *mmpL5* R202fs	Unknown	Missing
**HO-251**	2004	Ile67fs + *mmpL5* R202fs	Hypersusceptible	Peru
**LN-3755**	2004	Ile67fs + *mmpL5* R202fs	Hypersusceptible	Peru
**ERR2652986**	2004	Asp127Val	Unknown	Brazil
**PAR-044**	2005	Ile67fs + *mmpL5* R202fs	Hypersusceptible	Peru
**ERR023260**	2005.73*	Glu28Ala	Unknown	USA

Table S5: Number of estimated emergence (homoplasic) events for major *mmpR5* variants considered.

Variant	Predicted phenotype	L2	L4
Ile67fs	Resistant	11	7
Ile67fs + *mmpL5* Arg202fs*	Hypersusceptible	0	1
c-11a	Hypersusceptible	1	0
Asp5Gly	Susceptible	2	1
Met146Thr	Resistant	1	2
Leu40Val	Susceptible	0	1
Glu49fs	Resistant	2	7
Arg90Cys	Intermediate	1	1
Asp47fs	Resistant	7	1
Ala59Val	Resistant	1	1
Asn98Asp	Resistant	0	3
Leu117Arg	Resistant	2	4
Val1Ala	Resistant	2	0
Val20Ala	Intermediate	1	1
Arg156fs	Resistant	0	2
Asp141fs	Resistant	0	1
Gly121Arg	Resistant	1	1
Arg109Leu	Resistant	0	1
Arg96Gly	Resistant	0	4

Table S6: Summary of phylogenetic dating approaches applied to the L4 and L2 datasets. BactDating was run in both cases but failed to converge after 1e^7 or 3e^7 MCMC iterations for the L4 dataset. The subsampled BEAST2 runs highlighted yellow provided the highest likelihood following path sampling over all strict clock models and were run only on accessions with associated collection dates. These rates were applied to the maximum likelihood phylogenetic tree for temporal estimation of resistance emergence (main text Figure 2, see Methods).

Lineage	Analysis	Missing dates dropped		Using est dates	
		tmrca	sub rate (subs/site/year)	tmrca	sub rate (subs/site/year)
L2	BactDating 1e7	389 (-4201 - 963)	1.7e-07(1.5e-07 - 2.2e-07)	469 (-2749 - 894)	1.8e-07(1.6e-07 - 2.2e-07)
	BactDating 3e7	367 (-3257 - 987)	1.8e-07(1.5e-07 - 2.2e-07)	436 (-3252 - 903)	1.8e-07(1.58e-07 - 2.28e-07)
	BEAST2*, strict coalescent skyline	453 (-336 - 904)	7.7e-08 (4.9e-08 - 1.03e-07)	NA	NA
	BEAST2*, strict coalescent exponential	415 (-280 - 861)	7.4e-08 (4.9e-08 - 9.8e-08)	NA	NA
	BEAST2*, strict coalescent constant	465 (-218 - 877)	7.7e-08 (5.2e-08 - 1.01e-07)	NA	NA
	BEAST2*, relaxed coalescent skyline	509 (-505 - 1049)	8.2e-08 (4.8e-08 - 1.12e-07)	NA	NA
	BEAST2*, relaxed coalescent exponential	727 (-52 - 1217)	7.9e-08 (4.7e-08 - 1.1e-07)	NA	NA
	BEAST2*, relaxed coalescent constant	554 (-311 - 1088)	8.6e-08 (5.5e-08 - 1.17e-07)	NA	NA
L4	BEAST2*, strict coalescent skyline	292 (35 - 502)	7.1e-08 (6.2e-08 - 7.9e-08)	NA	NA
	BEAST2*, strict coalescent exponential	358 (116 - 557)	7e-08 (6.2e-08 - 7.9e-08)	NA	NA
	BEAST2*, strict coalescent constant	236 (-32 - 450)	6.9e-08 (6e-08 - 7.7e-08)	NA	NA
	BEAST2*, relaxed coalescent skyline	336 (-20 - 623)	7.3e-08 (6.2e-08 - 8.3e-08)	NA	NA
	BEAST2*, relaxed coalescent exponential	567 (278 - 794)	7.2e-08 (6.1e-08 - 8.2e-08)	NA	NA
	BEAST2*, relaxed coalescent constant	224 (-161 - 526)	6.9e-08 (5.9e-08 - 7.9e-08)	NA	NA

Table S7 [external excel document]: Inferred age of nodes and preceding nodes for L2 samples with *mmpR5* nonsynonymous variants. Cells are coloured as per phenotype annotations (see main text Figure 1-3). Presence of *mmpL5* variants is noted, as are MICs where available. TBProfiler resistance profiles as either "S" for susceptible, "RR" for rifampicin-resistant and "preXDR" for fluoroquinolone-resistant.

Table S8 [external excel document]: Predicted probability of bedaquiline resistance based on the amino acid properties of *mmpR5* variants following a machine learning predictive approach (see Methods and Supplementary Note 1).

Table S9: Precision, recall, F1, AUPRC, sensitivity and specificity scores for gradient-boosted tree classifier. Standard deviation was calculated across the 10 outer loops of the nested cross-validation protocol.

Metric	***Score***	***Standard dev.***
Precision	0.826	0.1
Sensitivity/Recall	0.754	0.1
F1	0.780	0.08
AUPRC	0.787	0.1
Specificity	0.633	0.3

Supplementary Note 1

Predicting bedaquline resistance using machine learning techniques

A gradient-boosted tree classifier was developed using the XGBoost API (v1.0.2)[20] to predict the phenotypic effect of *mmpR5* variants based on the amino acid properties of known resistant and susceptible variants. Mutations that confer resistance were used as the positive class in this binary classifier. Fifteen features were engineered based on the wild type and mutant residues of each mutation to include the amino-acid residue type, the polarity, charge, hydrophobic or hydrophilic status, molecular weight, and location including the 5'-3' position.

Only variants in *mmpR5* which have a demonstrated association to a bedaquiline-resistant or susceptible phenotype were used, resulting in 55 resistance and 27 susceptibility mutations. Model parameters were optimised to maximise the F1 score and model performance was estimated using a nested, stratified, 10 x 10 cross-validation procedure. AUPRC was used for model evaluation due to class imbalance in the dataset [18]. The trained model was then interpreted using TreeExplainer as part of the shap API (v0.35.0) (64) (**Additional file 2: Fig S10**) to allow inference of how each feature contributes to each prediction. All scripts used for the analysis are hosted on GitHub (https://github.com/cednotsed/TB-Bedaquiline-Resistance-Modelling.git). Predictions were also made using the Protein Variation Effect Analyzer (PROVEAN) via the online interface [19]. The final predicted probability of resistance and associated PROVEAN scores are provided in **Additional file 4: Table S8**.

Results

To assess properties associated to RAVs which may be useful predictors of the phenotypic effect of these unknown variants we applied a machine learning approach. A gradient-boosted tree classifier (XGBoost API (v1.02) was trained and optimised to determine if the amino acid

properties of *mmpR5* mutations associated to known bedaquiline resistance phenotypes can be used to predict the resistance status of mutations with no available phenotypic information.

Fifteen features were engineered based on the wild type and mutant residues of each mutation as follows. Two features represent the amino-acid residue of the wild type and of the mutant. Two features encode whether they are non-polar, polar, positively charged or negatively charged. Two features represent whether the wild type and the mutant residues are hydrophobic or hydrophilic, based on the hydrophobicity scale proposed by Janin[20]. Two features encode the molecular weight of wild type and mutant AA. Two features represent the change in charge or molecular weight from the wild type to mutant, where non-polar and polar residues are assumed to contribute a charge of zero. One feature represents the change in hydrophobicity, where a hydrophobic→hydrophilic residue change is coded as +1 and the reverse as -1. Three features represent mutations in the DNA-binding domain, in the dimerisation domain, and to the residues in contact with 2-stearoylglycerol. The last feature represented the 5'- 3' position of amino acid mutations.

Only variants in *mmpR5* which have a demonstrated association to a bedaquiline-resistant or susceptible phenotype were used, resulting in 55 resistance and 27 susceptibility mutations. In this instance we excluded frameshifts from the analysis as it is challenging to encode their physiochemical characteristics. Model parameters were optimised to maximise the F1 score and model performance was estimated using a nested, stratified, 10 x 10 cross-validation procedure. In this procedure, the model is trained and optimised on a subset of the data and the resultant model is evaluated on another subset of the data not used for training and optimisation. This process is then repeated 10 times and the model performance metrics are averaged to provide an assessment of how well the models perform on unseen data. AUPRC was used for

model evaluation due to class imbalance in the dataset[18]. The optimised model provided an area under the precision-recall curve (AUPRC) of 0.79 (**Additional file 4: Table S8**), suggesting that the physiochemical properties of mutations can be used to successfully differentiate between resistance and susceptibility phenotypes (**Additional file 2: Table S9**).

The trained model was then interpreted using TreeExplainer as part of the shap API (v0.35.0) (64). Each feature is assigned a SHAP value which represents the change in predicted probability score in each prediction when a feature is included or excluded from the model. Visualisation of the SHAP values in tandem with the feature values (**Addiitonal file 2: Fig S10**) allows inference of how each feature contributes to each prediction. The features of the models were then interpreted using SHAP values. As a measure of the relative importance and effect sizes for these predictor variables in the model, we calculated the mean absolute SHAP (MAS) values for all predictors, which measures the magnitude of the change in predicted probability of resistance caused by each variable in the model. For example, the variable 'WT hydrophobicity' (i.e., a measure of whether the wild-type amino acid is hydrophobic or not) had a MAS value of 7.8%, indicating that on average, this variable adds or subtract 7.8% to the probability of resistance predicted by the model. Via this approach, we found that mutations to polar and positively-charged residues, or of polar and positively-charged residues, and those to higher molecular weight amino acids are associated with resistance (MAS=2.1%, 4.5% and 2.5%, respectively). Conversely, mutations in the dimerisation or DNA-binding domains, transitions from negatively to positively charged residues, and mutations of hydrophobic residues, or mutations to hydrophobic residues are associated with susceptibility (MAS=4.8%, 2.2%, 2.6%, 7.8%, and 4.3%, respectively; **Additional file 2: Fig S10**). Overall, we demonstrate the promise of using the physiochemical characteristics of single mutations as input to machine learning models to predict their effects on drug susceptibility. Additionally,

we demonstrate the power of model interpretation approaches such as SHAP to derive biological insights from complex machine learning models.

In the absence of phenotypic data, machine learning approaches offer possibilities to predict the resistance status of given variants, and our small-scale analysis suggests the potential of such an approach. Our model uses general physiochemical characteristics of single non-synonymous mutations to infer information about drug susceptibility, which in principle allows us to predict the phenotypic effects of novel mutations that have not been characterised. However, even determining the phenotypic consequences of *mmpR5* variants that have previously been described is challenging as there are often only limited reports correlating MICs to genotypes. Moreover, at least four different methods are used to determine MICs, some of which do not have associated critical concentrations. Even where critical concentrations have been set, there is an overlap in MICs of isolates that are genetically wild type and those that have mutations likely to cause resistance [21]. Further, our machine learning modelling approach based on amino acid properties alone does not consider epistatic interactions that arise from the genetic background of isolates such as the mmpL5 frameshift mutations we observed. Future work aiming to include more genetic information in machine learning models, including frameshift mutations or different combinations of point mutations, may help better understand the correlations between genotype, protein function and phenotype, and may ultimately lead to better resistance predictions.

All scripts used for the analysis are hosted on GitHub (https://github.com/cednotsed/TB-Bedaquiline-Resistance-Modelling.git). Predictions were also made using the Protein Variation Effect Analyzer (PROVEAN) via the online interface[22]. The final predicted

probability of resistance and associated PROVEAN scores are provided in **Additional file 4: Table S8**.

References

1 Bloemberg, G. V. *et al.* Acquired Resistance to Bedaquiline and Delamanid in Therapy for Tuberculosis. *N Engl J Med* **373**, 1986-1988 (2015). https://doi.org:10.1056/NEJMc1505196

2 Xu, J. *et al.* Primary Clofazimine and Bedaquiline Resistance among Isolates from Patients with Multidrug-Resistant Tuberculosis. *Antimicrob Agents Chemother* **61** (2017). https://doi.org:10.1128/AAC.00239-17

3 Yang, J. S., Kim, K. J., Choi, H. & Lee, S. H. Delamanid, Bedaquiline, and Linezolid Minimum Inhibitory Concentration Distributions and Resistance-related Gene Mutations in Multidrug-resistant and Extensively Drug-resistant Tuberculosis in Korea. *Ann Lab Med* **38**, 563-568 (2018). https://doi.org:10.3343/alm.2018.38.6.563

4 Nimmo, C. *et al.* Bedaquiline resistance in drug-resistant tuberculosis HIV co-infected patients. *Eur Respir J* **55** (2020). https://doi.org:10.1183/13993003.02383-2019

5 Sonnenkalb, L. *et al.* Bedaquiline and clofazimine resistance in *Mycobacterium tuberculosis*: an in-vitro and in-silico data analysis. *Lancet Microbe* **4**, e358-e368 (2023). https://doi.org:10.1016/S2666-5247(23)00002-2

6 Ismail, N., Peters, R. P. H., Ismail, N. A. & Omar, S. V. Clofazimine Exposure In Vitro Selects Efflux Pump Mutants and Bedaquiline Resistance. *Antimicrob Agents Chemother* **63** (2019). https://doi.org:10.1128/AAC.02141-18

7 Villellas, C. *et al.* Unexpected high prevalence of resistance-associated *Rv0678* variants in MDR-TB patients without documented prior use of clofazimine or bedaquiline. *J Antimicrob Chemother* **72**, 684-690 (2017). https://doi.org:10.1093/jac/dkw502

8 Nimmo, C. *et al.* Population-level emergence of bedaquiline and clofazimine resistance-associated variants among patients with drug-resistant tuberculosis in southern Africa: a phenotypic and phylogenetic analysis. *Lancet Microbe* **1**, e165-e174 (2020). https://doi.org:10.1016/S2666-5247(20)30031-8

9 Ismail, N., Omar, S. V., Ismail, N. A. & Peters, R. P. H. Collated data of mutation frequencies and associated genetic variants of bedaquiline, clofazimine and linezolid resistance in Mycobacterium tuberculosis. *Data Brief* **20**, 1975-1983 (2018). https://doi.org:10.1016/j.dib.2018.09.057

10 Andries, K. *et al.* Acquired resistance of *Mycobacterium tuberculosis* to bedaquiline. *PLoS One* **9**, e102135 (2014). https://doi.org:10.1371/journal.pone.0102135

11 Karmakar, M., Rodrigues, C. H. M., Horan, K., Denholm, J. T. & Ascher, D. B. Structure guided prediction of Pyrazinamide resistance mutations in pncA. *Sci Rep* **10**, 1875 (2020). https://doi.org:10.1038/s41598-020-58635-x

12 Andres, S. *et al.* Bedaquiline-Resistant Tuberculosis: Dark Clouds on the Horizon. *Am J Respir Crit Care Med* **201**, 1564-1568 (2020). https://doi.org:10.1164/rccm.201909-1819LE

13 Zimenkov, D. V. *et al.* Examination of bedaquiline- and linezolid-resistant Mycobacterium tuberculosis isolates from the Moscow region. *J Antimicrob Chemother* **72**, 1901-1906 (2017). https://doi.org:10.1093/jac/dkx094

14 Pang, Y. *et al.* In Vitro Drug Susceptibility of Bedaquiline, Delamanid, Linezolid, Clofazimine, Moxifloxacin, and Gatifloxacin against Extensively Drug-Resistant Tuberculosis in Beijing, China. *Antimicrob Agents Chemother* **61** (2017). https://doi.org:10.1128/AAC.00900-17

15 Guglielmetti, L. *et al.* Long-term outcome and safety of prolonged bedaquiline treatment for multidrug-resistant tuberculosis. *Eur Respir J* **49** (2017). https://doi.org:10.1183/13993003.01799-2016
16 Martinez, E. *et al.* Mutations associated with in vitro resistance to bedaquiline in *Mycobacterium tuberculosis* isolates in Australia. *Tuberculosis (Edinb)* **111**, 31-34 (2018). https://doi.org:10.1016/j.tube.2018.04.007
17 Rancoita, P. M. V. *et al.* Validating a 14-Drug Microtiter Plate Containing Bedaquiline and Delamanid for Large-Scale Research Susceptibility Testing of *Mycobacterium tuberculosis*. *Antimicrob Agents Chemother* **62** (2018). https://doi.org:10.1128/AAC.00344-18
18 Saito, T. & Rehmsmeier, M. The Precision-Recall Plot Is More Informative than the ROC Plot When Evaluating Binary Classifiers on Imbalanced Datasets. *Plos One* **10** (2015). https://doi.org:ARTN e0118432
10.1371/journal.pone.0118432
19 Baele, G. *et al.* Improving the accuracy of demographic and molecular clock model comparison while accommodating phylogenetic uncertainty. *Mol Biol Evol* **29**, 2157-2167 (2012). https://doi.org:10.1093/molbev/mss084
20 Janin, J. Surface and inside Volumes in Globular Proteins. *Nature* **277**, 491-492 (1979). https://doi.org:DOI 10.1038/277491a0
21 Organization, W. H. Technical report on critical concentrations for
TB drug susceptibility testing of medicines used in the treatment of drug-resistant TB. (2018).
